# Eliminating the Neglected Tropical Diseases: Translational Science and New Technologies

**DOI:** 10.1371/journal.pntd.0003895

**Published:** 2016-03-02

**Authors:** Peter J. Hotez, Bernard Pecoul, Suman Rijal, Catharina Boehme, Serap Aksoy, Mwelecele Malecela, Roberto Tapia-Conyer, John C. Reeder

**Affiliations:** 1 Sabin Vaccine Institute and Texas Children’s Hospital Center for Vaccine Development, National School of Tropical Medicine, Baylor College of Medicine, Houston, Texas, United States of America; 2 Drugs for Neglected Diseases Initiative (DNDi), Geneva, Switzerland; 3 Drugs for Neglected Diseases Initiative (DNDi), Delhi, India; 4 Foundation for Innovative new Diagnostics (FIND), Geneva, Switzerland; 5 Yale School of Public Health, Department of Epidemiology of Microbial Diseases, New Haven, Connecticut, United States of America; 6 National Institute for Medical Research, Dar es Salaam, Tanzania; 7 Carlos Slim Foundation, Mexico City, Mexico; 8 UNICEF/UNDP/ World Bank/WHO Special Programme for Research and Training in Tropical Diseases (TDR), Geneva, Switzerland; New York Blood Center, UNITED STATES

## Abstract

Today, the World Health Organization recognizes 17 major parasitic and related infections as the neglected tropical diseases (NTDs). Despite recent gains in the understanding of the nature and prevalence of NTDs, as well as successes in recent scaled-up preventive chemotherapy strategies and other health interventions, the NTDs continue to rank among the world’s greatest global health problems. For virtually all of the NTDs (including those slated for elimination under the auspices of a 2012 London Declaration for NTDs and a 2013 World Health Assembly resolution [WHA 66.12]), additional control mechanisms and tools are needed, including new NTD drugs, vaccines, diagnostics, and vector control agents and strategies. Elimination will not be possible without these new tools. Here we summarize some of the key challenges in translational science to develop and introduce these new technologies in order to ensure success in global NTD elimination efforts.

## Overview of the Neglected Tropical Diseases

The modern framework for considering a group of parasitic and related infections as neglected tropical diseases (NTDs) was put forward by the World Health Organization (WHO) together with a series of key policy papers published by a community of scientists in the early years following the Millennium Development Goals (MDGs) [1–5]. An original list of more than a dozen NTDs—identified by their disproportionate impact on the world’s poor and for their ability to cause poverty through deleterious effects on worker productivity, child development, and the health of girls and women [2,6]—was subsequently expanded by the WHO to include 17 diseases [7]. Through the Global Burden of Disease Study 2010 (GBD 2010), new information on the prevalence of the NTDs confirms that they rank as the most common afflictions of the world’s poor (led by ascariasis, trichuriasis, and hookworm with more than 400 infections each, followed by schistosomiasis), with practically every person living in conditions of extreme poverty infected by at least one NTD [8].

**Fig 1:**
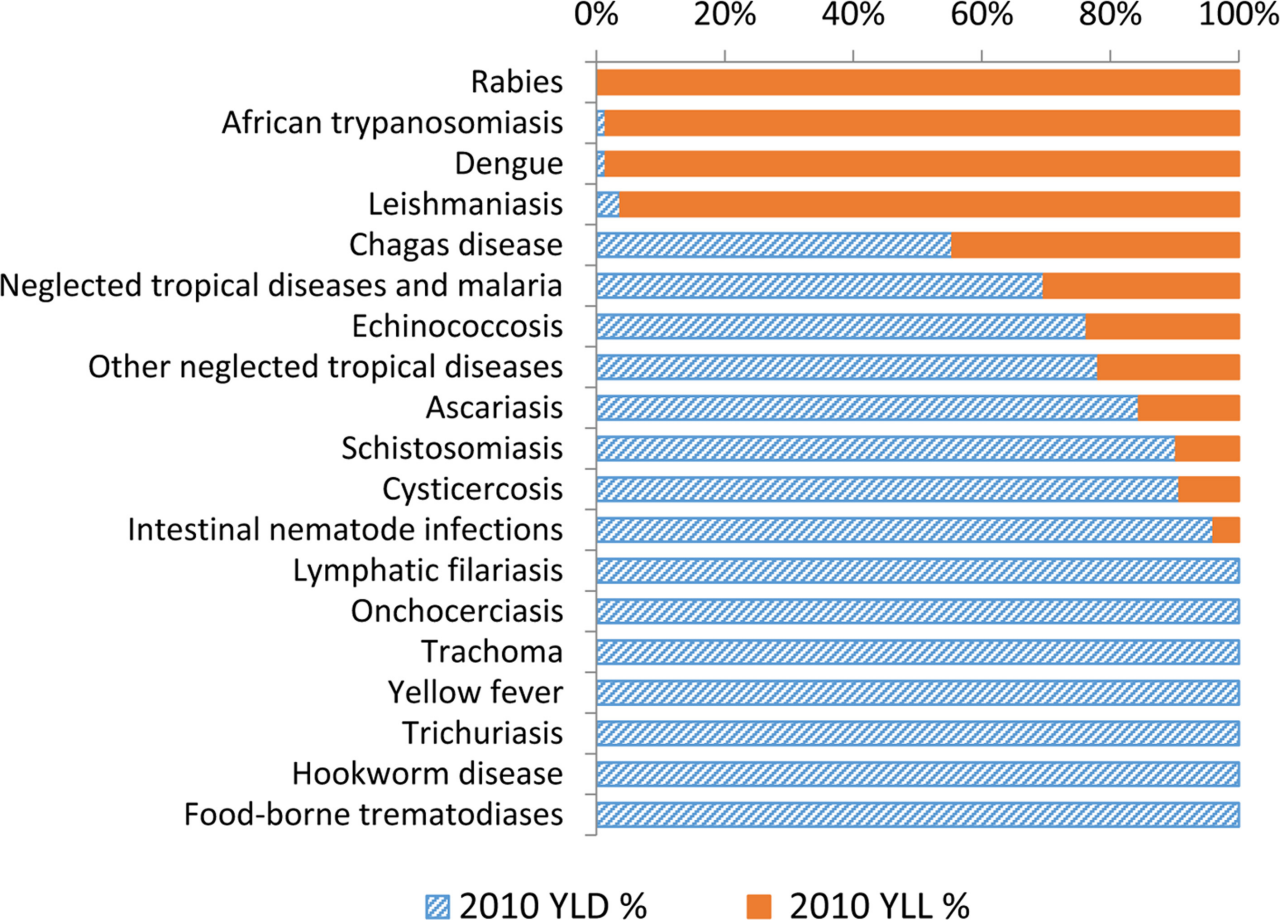
Fractions of years lived with disability (YLD) and years of life lost (YLL) due to premature death (as components of disability adjusted life years [DALYs]) for each of the NTDs. Also included in this graph are “other NTDs.” Figure previously published in Hotez et al., 2014 [8].

The GBD 2010 also finds that while some NTDs such as rabies, African sleeping sickness, and visceral leishmaniasis are lethal diseases, most of the NTDs are highly disabling, and many are chronic in nature (Fig 1) [8].

The leading NTDs in terms of disability adjusted life years (DALYs) lost include schistosomiasis, leishmaniasis, and hookworm infection [8]. Additional information has emphasized the social stigmatizing effects from the NTDs and their poverty-promoting features, and their links to regional and global conflict. These elements of the NTDs demand that they remain front and center as we advance to a new set of “sustainable development goals” (SDGs).

Since 2006, a major approach to controlling the NTDs has relied on mass treatment with one or more essential drugs, mostly donated by the major multinational pharmaceutical companies [2–7]. Initially known as the “rapid impact” package—containing up to four drugs that target the three major soil-transmitted helminth infections (albendazole or mebendazole), schistosomiasis (praziquantel), lymphatic filariais (ivermectin or diethylcarbamazine citrate), onchocericiasis (ivermectin), and trachoma (azithromycin) [2,3]—more than one billion people have now received at least one of these essential NTD medicines. The WHO has designated this approach as “preventive chemotherapy” because for some diseases such as lymphatic filariasis (LF) and trachoma, mass treatment is leading to the interruption of transmission and disease elimination [9]. Moreover, preventive chemotherapy has been shown to result in spillover or collateral public health benefits, including the potential control or elimination of neglected skin diseases such as scabies and yaws [10]. It is estimated that, overall, approximately 36% of the global population living in poverty and requiring preventive chemotherapy actually received treatment in the year 2012 [11], highlighting the urgent need for greater investment worldwide. Based on findings that an unexpectedly high percentage of NTDs occurs among the poor who live in the wealthier group of 20 (G20) nations [12], there is urgency to pressure the G20 to invest more heavily in their own NTDs [13].

Two important post-MDGs policy documents for NTD control and elimination include the 2012 London Declaration on NTDs—which reaffirms a commitment by the multinational pharmaceutical companies to continue donating essential NTD medicines and invest more seriously in research and development (R&D) for new tools [14]—and a 2013 World Health Assembly resolution on the NTDs (WHA66.12) [9]. Both the London Declaration and WHA66.12 highlight opportunities to also eliminate NTDs such as dracunculiasis, human African trypanosomiasis (HAT), leprosy, onchocerciasis in the Americas, and schistosomiasis in China, with more than 74 countries worldwide implementing national NTD master plans [9,11].

## New Technologies for Improving Patient Care and Achieving Disease Elimination

**Fig 2:**
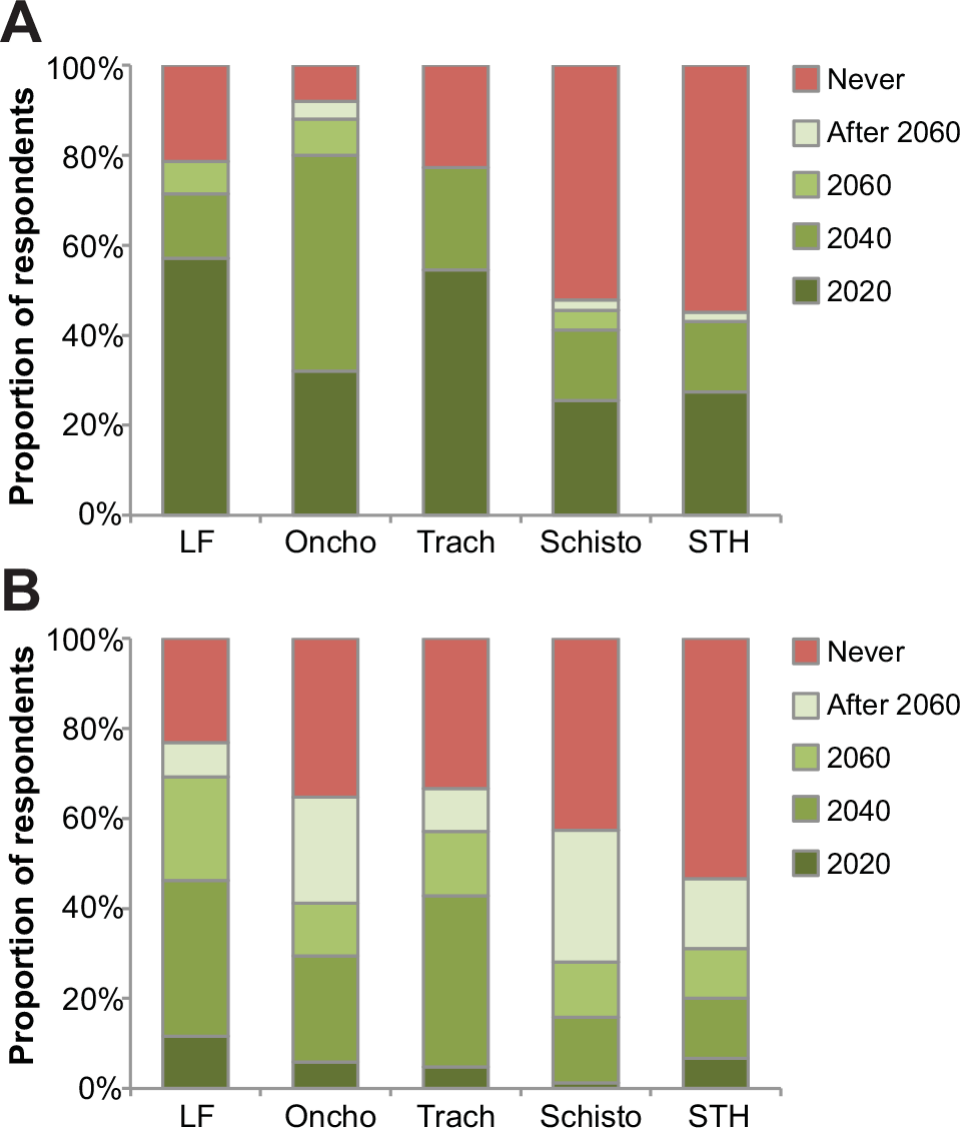
Timeline for elimination (top) and eradication (bottom) of targeted NTDs by preventive chemotherapy. Figure previously published in Keenan et al., 2013 [16].

Critical disease-specific technologies—drugs, vaccines, diagnostics, and vector control agents—are currently available to facilitate the control or elimination of many of the world’s NTDs [14]. There is particular enthusiasm for the global elimination of the NTDs, lymphatic filariasis, trachoma, and yaws through preventive chemotherapy and mass treatment using currently available drugs. However, for most of the 17 NTDs, drug, vaccine, diagnostic and vector control technologies are imperfect and have limited use because of their toxicities, inadequate efficacies, or because they do not prevent reinfection [15]. For example, a recent survey of almost 400 NTD experts concluded that currently available technologies will not eliminate soil-transmitted helminth (STH) infections or schistosomiasis, two of the most ubiquitous NTDs being targeted (Fig 2) [16].

There is, therefore, an essential need for new or improved technologies to control these diseases [15].

Whether STH infections, schistosomiasis, and onchocerciasis (now targeted by preventive chemotherapy) could be eliminated without new and additional tools has also generated debate. For instance, a recent analysis based on modeling indicates that interruption for STH infections using current mass treatment approaches with benzimidazole anthelminthics may be feasible in areas of low intensity transmission and where strong health systems are in place [17]. For schistosomiasis, efforts are underway in Zimbabwe to evaluate whether mass treatment with praziquantel, together with either behavioral changes or snail control might affect elimination [18]. For onchocerciasis, some modeling indicates that elimination through ivernectin preventive chemotherapy may be achievable if it were continued up to 2030 or even 2040 [19]. Therefore, there is a benefit to increased dialogue between the community of researchers engaged in modeling NTD elimination scenarios and those who are developing next generation tools and products, specifically drugs, diagnostics, vaccines, and vector control agents.

Because many of these NTDs either mostly or exclusively affect people living in poverty, they are being developed primarily by nonprofit product development partnerships focused on specific NTD drugs (e.g., Drugs for Neglected Diseases Initiative [DNDi] and PATH), diagnostics (e.g., Foundation for Innovative New Diagnostics [FIND] and PATH), vaccines (e.g., PATH, Infectious Disease Research Institute [IDRI], and Sabin Vaccine Institute), and vector control agents (e.g., Innovative Vector Control Consortium [IVCC]), working together with industry (including both multinational companies and industrial organizations in developing countries) and academic organizations.

## New NTD Drugs

The last decade has seen incremental improvements in the development of NTD treatments. An analysis of the neglected disease R&D pipeline from 2000 to 2011 found that most advances entailed repurposing or reformulating existing drugs [20]. Entirely new chemical entities (NCEs) are still needed to improve patient care and meet current elimination targets.

### Drugs for Kinetoplastid Infections

Until recently, treatment options for kinetoplastids were toxic and ill-adapted. Increased interest in drug discovery is slowly transforming the landscape.

- Human African trypanosomiasis (HAT): An oral short-course treatment for early and advanced stages of the disease and a rapid diagnostic test, are required for sustainable elimination of the disease. While nifurtimox-eflornithine combination therapy, introduced in 2009, has improved case management, it is not practical for large-scale “test and treat” efforts [21]. Two NCEs are currently undergoing clinical evaluation as oral therapies: fexinidazole (Phase IIb/III trials), and the oxaborole SCYX-7158 as a one-dose treatment (Phase I). Several candidates are in lead optimization [22–25].
- Chagas disease: Only two drugs exist: benznidazole and nifurtimox. Progress has been made in child-adapted dosage forms (pediatric benznidazole registered in 2011), but the scaling up of treatment in general is far too slow. Posaconazole and a prodrug of ravuconazole (E1224) have shown some promise but are not effective as monotherapies. Currently, shorter courses of benznidazole, as well as E1224 combined with benznidazole, are being evaluated [26].
- Leishmaniasis: Treatment has improved over the last decade in Asia, with the development of liposomal amphotericin B, paromomycin, and miltefosine. Nevertheless, adapted treatments are lacking for Africa and Latin America. Oral therapies are being pursued for use in drug combinations to improve treatment for visceral leishmaniasis and coinfection with HIV [27,28]. For cutaneous leishmaniasis, topical creams are currently in clinical trials [29,30].

To ensure that new compounds and back-up molecules are available for kinetoplastids, high-throughput screening of core diversity libraries from several pharmaceutical companies is being conducted. Promising leads from the nitroimidazole and oxaborole series for both leishmaniasis and Chagas are being evaluated.

### Drugs for LF and Onchocerciasis

Ironically, the success of mass treatment programs for filarial infections may have undermined support for R&D [31,32]. Nonetheless, promising areas of research are genome sequencing of filarial helminths, the development of drugs against Wolbachia endosymbionts, active screening of repurposing libraries, and specific development projects for new macrofilaricidal drug candidates (e.g., emodepside and flubendazole). Elimination targets will be difficult to achieve without a macrofilaricide.

### Drugs for Bacterial and Viral NTDs

There are no drugs in development for leprosy, trachoma, and yaws due to the availability of antibiotics. For Buruli ulcer, experimental substitutions of other antibiotics are being evaluated. One nucleoside inhibitor has completed Phase I for dengue fever. For Ebola virus disease, the West African epidemic has given rise to long overdue attention to and resources for new treatment, though progress remains far too slow and recruitment for trials has been complicated by the otherwise good news that patient numbers are decreasing.

### Key Challenges

After years of neglect, NTD drug development efforts are finally intensifying, though not sufficiently. New nonprofit initiatives and increased funding have improved the prospects for NTD drug R&D somewhat, but efforts are still too fragmented and ad hoc. New R&D incentive mechanisms and innovative financing instruments, as well as improved global priority-setting and coordination, will be critical to ensure that treatments are brought out of the pipeline and put into the hands of neglected patients [33].

## New NTD Vaccines

The 2014 West African Ebola virus epidemic identified serious gaps in our abilities to advance in a timely manner the development and advanced clinical testing of vaccines to combat the NTDs. Most of these deficiencies were unrelated to the feasibility of making Ebola vaccines, which was established in nonhuman primates almost a decade prior to the outbreak. Instead, our technical abilities outpaced the social, political, economic, and financial institutions for making vaccines for the extremely poor. As a result of absent financial incentives, market failures, and other socioeconomic forces, we have no licensed NTD vaccines except for rabies, while for the other NTDs only candidate dengue virus vaccines are currently in Phase II and III advanced clinical development. The reason for this situation is mostly that there is a small but substantial market for rabies and dengue biotechnologies among the middle- and high-income countries.

### Anthelminthic Vaccines

As pointed out previously, it will not be feasible to achieve elimination targets for most helminth infections with preventive chemotherapy alone [16]. Several anthelminthic vaccines for hookworm and other soil-transmitted helminth infections, as well as for schistosomiasis and onchocerciasis, are in varying stages of development. A bivalent recombinant human hookworm vaccine is in Phase I clinical testing in endemic areas of Brazil and Gabon through a HOOKVAC consortium of European partners and the Sabin Vaccine Institute product development partnership (PDP) [34,35], while vaccine antigens for ascariasis and trichuriasis are undergoing preclinical testing [36]. Several vaccines for both urogenital and intestinal schistosomiasis are also in Phase I trials (or at an advanced stage of preclinical development) or later stages of clinical development [37–41]. Finally, through The Onchocerciasis Vaccine for Africa (TOVA) initiative, a new vaccine may soon enter Phase I testing [42]. Most of these anthelminthic vaccines could be used in conjunction with anthelminthic drugs in programs of “vaccine-linked chemotherapy” in order to prevent reinfection following mass drug administration (MDA) [43]. Two transmission-blocking veterinary vaccines are also being developed for zoonotic helminth infections to prevent taeniasis and Asian schistosomiasis [44,45].

### Kinetoplastid Vaccines

Several candidate leishmaniasis vaccines are under development both for the visceral and cutaneous forms of this disease, by the Infectious Diseases Research Institute (IDRI) PDP, the Sabin PDP, and two European consortia [46–49]. A therapeutic Chagas disease vaccine is also being developed by the Sabin PDP in collaboration with the Carlos Slim Foundation [50], in parallel with other approaches [51,52].

### Key Challenges

Despite published studies showing the cost-effectiveness or even cost-savings of NTD vaccines [53–56], the major international agencies charged with advancing and introducing new vaccines—such as WHO and Gavi, the Vaccine Alliance—have been slow to adopt and promote them. There are multiple reasons for this situation, including the lack of engagement of major multinational pharmaceutical companies and the fact that NTD vaccines might prevent disability rather than under-five childhood mortality. Another major concern is the significant potential costs attached to advanced clinical development for NTD vaccines in the absence of surrogate correlates of protection. Overcoming these challenges could require developing human challenge models to obtain a “quick read” on vaccine efficacy—an approach that is being pursued for hookworm infection and possibly leishmaniasis—in order to reduce investment risk. Other risk-reducing activities could include combining NTD vaccines with those for malaria or other priority diseases in order to make investments more attractive to the industry and partnering with Developing Country Vaccine Manufacturing Networks (DCVMs) for industrial-scale production.

## New NTD Diagnostics

A major challenge to meeting the goals of the London Declaration is the lack of readily available, easy-to-use, reliable, and low-cost diagnostic tools to identify infected patients, confirm cure, monitor the impact of mass treatment programs, and watch for disease re-emergence. Thirteen of the 17 NTDs (as currently defined by WHO) lack essential diagnostics, including seven of the 10 in the London Declaration. While recently there has been greater recognition of the role of diagnostics in meeting 2020 goals, funding commitments are lacking [57,58].

For many of the diseases that need to be managed on a case-by-case basis, notably Chagas disease, HAT, leprosy, visceral leishmaniasis, and Buruli ulcer, the diagnostic pathway is complex, and for some, confirmatory diagnosis requires invasive procedures not usually available at the community level. Investment in the development of appropriate diagnostics will make possible early and comprehensive case detection. For diseases controlled through mass treatment programs, we need tests to identify and map the populations requiring treatment and those in which transmission has been successfully interrupted. New diagnostics are also critically important for monitoring elimination progress and ongoing surveillance [59].

The R&D pipeline for NTD diagnostics overall is not sufficiently robust. For some, such as HAT, schistosomiasis, and onchocerciasis, substantial progress has been made in the development of new point-of-care tests, but they have not yet been widely implemented in endemic countries [60–62]. However, we are still at the biomarker discovery stage for new diagnostics for diseases such as Chagas and soil-transmitted helminthiases. For others, there is very little on the development horizon and no funding available at all. The example of Buruli ulcer, which had nothing new in the diagnostic pipeline until very recently, shows how even limited investment can catalyze R&D progress, with rapid tests expected in the next two years, pending adequate funding.

### Key Challenges

In order to foster the development and widespread implementation of new diagnostic tools for NTDs, creative solutions are needed, including the formation of a representative diagnostics coalition that includes countries, researchers, developers, and policymakers to define and prioritize NTD diagnostic needs; improvement of the business case for manufacturers to foster investment by promoting the use of existing and emerging diagnostics platforms [63]; and development of tests to meet multiple diagnostic needs simultaneously. For example, a test that detects both malaria and HAT is now being explored and could become an excellent tool for malaria diagnosis while maintaining surveillance for HAT in an elimination setting. In addition, triaging tests that differentiate various causes of fever or classes of pathogens (bacterial versus viral versus parasitic) could serve multiple purposes: patient management for NTDs such as dengue and leishmaniasis, rational use of drugs to prevent the emergence of drug resistance, and disease surveillance.

The London Declaration has already generated important momentum in the development of new tools to control and eliminate NTDs. The next step is to capitalize on near-term diagnostic successes, such as the HAT rapid test, to build the investment case in support of innovative solutions and ensure that funding commitments match the needs.

## New NTD Vector Control Agents and Strategies

Many of the NTDs are transmitted by insects, including mosquitoes, tsetse flies, reduviid bugs, black flies, and sand flies, which continue to plague the health of millions of people worldwide. In many instances, disease control can be effectively achieved by reducing or eliminating vector populations. Furthermore, vector control is necessary where domestic or wild animal reservoirs contribute to the maintenance of transmission. Current disease control and elimination programs are centered on preventive chemotherapy, with insufficient focus on vector control and source-reduction strategies. It is evident, however, that without enhanced vector control, diseases like LF will take longer to eliminate. In several countries in Africa, the Anopheles mosquito that spreads malaria is also the same mosquito that spreads LF, so there may be opportunities to link NTD- and malaria-control strategies.

Vector control has traditionally been achieved via the application of insecticides, particularly against vectors of Chagas disease in the Americas [64]. Chemical agents used for insecticides typically function by either exhibiting direct toxicity against larvae or adults, or by luring insects away from human hosts and into baited traps. However, insecticides are expensive and their sustained use has been challenged by the rapid emergence of resistance in many insect species, including mosquitoes and black flies [65,66].

Research on the molecular genetics of insecticide resistance will help identify the mechanisms that mediate resistance in field populations. This information can then be exploited to help develop efficient diagnostic assays and management strategies to combat the emergence of resistance [67]. Furthermore, the development of longer-lasting insecticide formulations and more efficacious larvicides [68], coupled with targeted application practices, could increase the efficacy and substantially reduce the cost of these chemical-based methods [69].

Advances in functional genomics research, in conjunction with the growing abundance of insect and pathogen genomic data, now set the stage for the development of novel vector control methods [70,71]. Research into the genetic basis of vector competence (the ability of insect hosts to transmit specific pathogens) has identified molecular mechanisms that contribute to natural insect resistance against disease-causing pathogens. Experimentally derived approaches that fortify hostile disease vector responses can prevent the development of pathogens in their insect hosts and reduce or block disease transmission to the mammalian hosts. In this regard, efforts have focused on modifying the symbiotic bacteria that reside naturally within insect vectors and alter important host physiologies and affect vector competence [72–74].

In addition to methods modifying vector competence, transgenics-based approaches that reduce insect population densities are also attractive. One such approach, designated “Release of Insects with Dominant Lethality” (RIDL), involves releasing genetically engineered male insects that breed with wild females and produce dead progeny [75], or flightless females [76]. Finally, the expanding genomics and functional biology knowledge related to insect smell, or “olfactory physiology,” provides a foundation for the development of novel baits that can enhance the efficacy of targets and traps in the case of tsetse flies [77] and mosquitoes [78,79]. Additionally, relevant technologies can be applied to modify vector host preference and olfaction [80].

### Key Challenges

The pipeline for new insecticides is limited. Thus, it is imperative to retain the efficacy of existing chemicals. Some of the new, innovative strategies highlighted above, which are designed to reduce human–vector contact or inhibit vector competency, represent promising new strategies. Furthermore, these approaches have the potential to reduce the cost of vector control related to NTDs. Additional new research that aims to improve on existing control tools—including olfaction technologies to alter mosquito behavior or enhanced targets and traps used for tsetse control [81,82]—will also be highly beneficial. The ongoing evaluation of such technologies in endemic settings, together with programs of community advocacy and education could ensure their eventual acceptability.

## Integrating New Tools into Elimination Strategies

Eliminating diseases requires not only the right tools, but also the system to deliver them in a timely and efficient manner. This requires a different type of innovation that is dependent on local capacity and implementation science, where we move from the question of “can this work” to “how can it work here?” We have learned that new tools will not deliver themselves. In order for the tools to be meaningful and have an impact, understanding the local context in which the tools will work is key. There is also a need to model how these new tools would promote global elimination efforts for each of the major NTDs.

Discovery science for drug and diagnostics development is often conducted on a global stage, but increasingly we need to recognize that R&D must also consider a local context that involves scientists, clinicians, regulators, and health ministries in disease-endemic countries. Moreover, implementation science must have local context. This requires that multidisciplinary teams including policymakers, social scientists, health administrators, and communication scientists work together in new ways [83,84].

A clear message of the recent publication “WHO World Health Report: Research for Universal Health Coverage” [85] is that all countries must become not only research users but research producers [86]. For this to happen, there must be strengthening of capacity to conduct research and translate this into policy in the disease-endemic countries themselves. For the last 40 years, the Special Programme for Research and Training in Tropical Diseases (TDR) has been doing just this and has learned a number of important lessons to be considered as we move forward [87].

Defining system bottlenecks and research priorities for addressing them at a country level, and engaging community support, have been critical factors in some of the most successful control programs [88], such as the undisputed success of the development of community-based onchocerciasis control strategies, which treated over 100 million people in 19 African countries as of the end of 2012 [89,90].

Most critical, though, has been long-term commitment to developing individual and institutional capacity in the affected countries [91]. The most important component of research is the researcher, and with endemic country researchers combining local knowledge with quality science, there is great hope for integrating new tools effectively into elimination programs. Let us not forget to invest in this essential part of the research and development cycle.

**Key Learning Points**

- Several new small-molecule drugs are being advanced for the major kinetoplastid infections: human African trypanosomiasis (HAT), Chagas disease, and leishmaniasis, as well as new macrofilaricides and drugs for Buruli ulcer, dengue, and Ebola virus infection. These new drugs will need to be integrated into case detection and management and other control or elimination programs.
- Vaccines for human hookworm infection, schistosomiasis, leishmaniasis, and Ebola virus infection are in clinical trials, while transmission blocking vaccines targeting zoonotic reservoir hosts are in advanced development for taeniasis (cysticercosis) and Asian schistosomiasis.
- There is an urgent need for new point-of-care (POC) diagnostics for most of the NTDs, in addition to tests that meet multiple diagnostic needs simultaneously, such as a test that detects both malaria and HAT.
- Control of vector-transmitted NTDs can be realized through vector reduction or elimination. Many of these approaches, however, rely on the use of chemicals. The presence of high levels of resistance in insects to these chemicals is compromising the major tool available for vector control. There is an urgent need for the development of new chemicals and the need to preserve the efficacy of those currently available. Advances in the genomics and molecular genetics of vectors, together with new transgenic and paratransgenic methodologies, are leading to new and innovative approaches to vector control.
- There is a need to shape public policies for the introduction of these new technologies and ensure they meet World Health Organization (WHO) goals for universal health coverage. In parallel, we need to strengthen capacity for research and development in disease-endemic countries.

**Top Five Papers**

1. Hotez PJ, Alvarado M, Basanez MG, Bolliger I, Bourne R, et al. (2014) The global burden of disease study 2010: interpretation and implications for the neglected tropical diseases. PLoS Negl Trop Dis 8: e2865.
2. World Health Organization (2015) Investing to overcome the global impact of neglected tropical diseases: Third WHO report on neglected tropical diseases. Geneva. 191 p.
3. Keenan JD, Hotez PJ, Amza A, Stoller NE, Gaynor BD, et al. (2013) Elimination and eradication of neglected tropical diseases with mass drug administrations: a survey of experts. PLoS Negl Trop Dis 7: e2562.
4. Reeder JC, Guth JA (2015) What have we learned from 40 years of supporting research and capacity building? PLoS Negl Trop Dis 9: e3355.
5. BIO Ventures for Global Health (2015) Global Health Primer. http://www.bvgh.org/Current-Programs/Neglected-Disease-Product-Pipelines/Global-Health-Primer.aspx. Accessed February 1, 2015.
